# Selection and structural bases of potent broadly neutralizing antibodies from 3-dose vaccinees that are highly effective against diverse SARS-CoV-2 variants, including Omicron sublineages

**DOI:** 10.1038/s41422-022-00677-z

**Published:** 2022-06-07

**Authors:** Lei Wang, Wangjun Fu, Linlin Bao, Zijing Jia, Yuxia Zhang, Yunjiao Zhou, Wei Wu, Jianbo Wu, Qianqian Zhang, Yidan Gao, Kang Wang, Qiao Wang, Chuan Qin, Xiangxi Wang

**Affiliations:** 1 CAS Key Laboratory of Infection and Immunity, National Laboratory of Macromolecules, Institute of Biophysics, Chinese Academy of Sciences, Beijing, China; 2 University of Chinese Academy of Sciences, Beijing, China; 3 Key Laboratory of Human Disease Comparative Medicine, Chinese Ministry of Health, Beijing Key Laboratory for Animal Models of Emerging and Remerging Infectious Diseases, Institute of Laboratory Animal Science, Chinese Academy of Medical Sciences and Comparative Medicine Center, Peking Union Medical College, Beijing, China; 4 Changping Laboratory, Beijing, China; 5 Key Laboratory of Medical Molecular Virology (MOE/NHC/CAMS), Shanghai Institute of Infectious Disease and Biosecurity, Shanghai Frontiers Science Center of Pathogenic Microbes and Infection, School of Basic Medical Sciences, Shanghai Medical College, Fudan University, Shanghai, China

**Keywords:** Electron microscopy, Immunology

Dear Editor,

The severe acute respiratory syndrome coronavirus 2 (SARS-CoV-2), belonging to the lineage B of the genus Betacoronavirus in the Coronaviridae family, is the causative agent of the COVID19 pandemic,^1^ which has lasted for over two years, resulting in unprecedented public health and socioeconomic crisis. Several SARS-CoV-2 variants of interest (VOIs) as well as variants of concern (VOCs), including the Alpha (B.1.1.7), Beta (B.1.351), Gamma (P.1), Delta (B.1.617.2) and the most recently identified Omicron sublineages (BA.1, BA.1.1, BA.2 and BA.3), have emerged at different time points over the course of the pandemic, leading to the resurgence of outbreaks across the world. Among these variants, Omicron, with a dramatically increased infectivity and enhanced transmissibility, is rapidly replacing the Delta variant and is poised to become a predominately prevalent strain in more than 110 countries since it was first reported in South Africa at the end of 2021.^2^ As a newly characterized VOC, Omicron was classified as the fifth VOC by the World Health Organization (WHO), with over 50 amino acid mutations, 37 of which are located in the spike (S) protein. The constellation of mutations allowed Omicron to evade immune responses induced by vaccination or natural infections and confer resistance to most of the commercially developed neutralizing antibodies (NAbs),^3–6^ making this variant more dangerous than its predecessors. In previous studies, we isolated approximately 400 monoclonal antibodies from participants who had received 3 doses of inactivated vaccine (Coronavac).^3,7^ One subset of these exhibited broad and potent neutralization activities against SARS-CoV-2 variants.^3^ The top 3 most potent NAbs (XGv051, XGv264, and XGv286) against Omicron sublineages were selected for immunogenic and structural analysis, which is instructive for structure-based broad-spectrum vaccine design.

Biolayer interferometry (BLI) measurements showed that these three antibodies bound tightly to wild-type (WT) and other four VOCs at sub-nM levels ranging from 0.004 to 0.5 nM, but exhibited a 5–15-fold decrease in binding affinity to Omicron (Fig. 1a; Supplementary information, Fig. S1). To evaluate the potency and breadth of neutralizations mediated by these three antibodies, we firstly performed the neutralization assays using pseudoviruses bearing the S of WT or from five VOCs. The half-maximal inhibitory concentrations (IC_50_) of the three NAbs against WT and five VOCs ranged from 0.002 to 0.039 μg/mL (Fig. 1a; Supplementary information, Fig. S2 and Table S3). Although all three NAbs exhibited potent neutralizing activities against WT and VOCs, the neutralizing potency of the NAbs varied slightly depending on the type of the variant. Notably, XGv051, XGv264, and XGv286 neutralized Omicron BA.1 with strikingly low IC_50_ values of 0.003, 0.004 and 0.006 μg/mL, respectively, albeit with slightly reduced activities against BA.2 and BA.3, indicating that these NAbs exhibit comparable or even better neutralization potencies against the most alarming variant when compared to WT or other VOCs, respectively (Fig. 1a; Supplementary information, Fig. S2). Experiments repeated using authentic virus, including WT and four circulating VOCs, showed similar neutralization patterns with IC_50_ values ranging from 0.003 to 0.05 μg/mL (Fig. 1a).

**Fig. 1:**
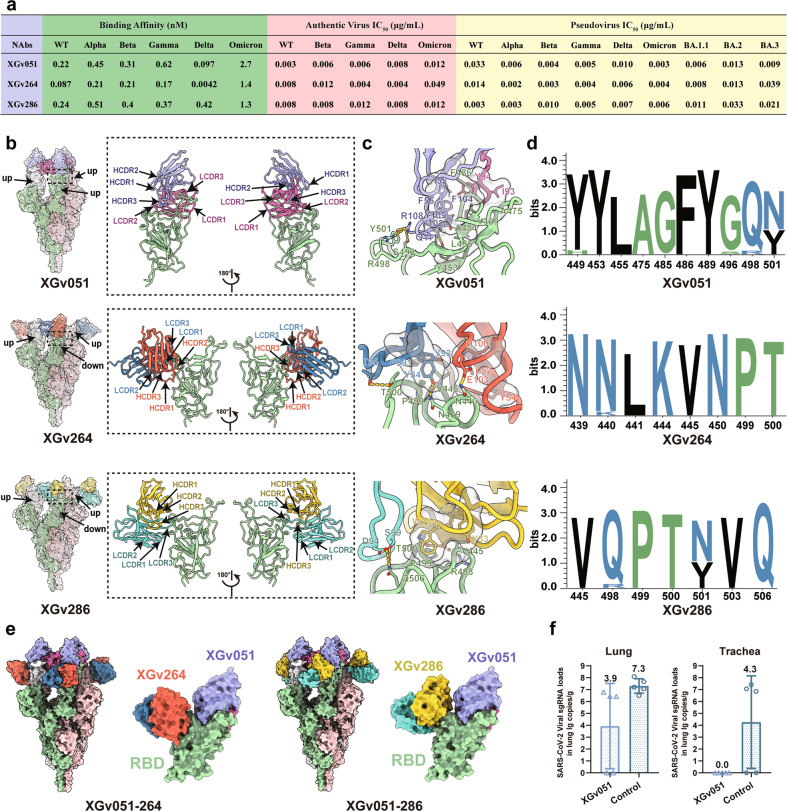
Structural and functional characterizations of XGv051, XGv264, and XGv286. **a** A summary of binding affinity and neutralization activity of three antibodies against SARS-CoV-2. Binding kinetics of XGv051, XGv264 and XGv286 to WT SARS-CoV-2 or five VOCs are measured by BLI. IC_50_ values for XGv051, XGv264 and XGv286 against WT or VOCs are listed. **b** Surface representations of the structures of SARS-CoV-2 Omicron S trimer in complex with XGv051, XGv264 and XGv286 with different colors for each S monomer (light green, pink and white), XGv051(light chain: deep pink; heavy chain: purple), XGv264 (light chain: blue; heavy chain: red) and XGv286 (light chain: green; heavy chain: gold). The enlarged panels on the right show views for binding interfaces to illustrate the binding modes of these three antibodies. **c** Details of the interactions between these three antibodies and SARS-CoV-2 Omicron RBD. Residues involved in the formation of hydrophobic patches and hydrogen bonds are shown as transparent surface and labeled, respectively. Color scheme is the same as in **b**. **d** Analysis of sequence conservation on residues involved in antibodies binding. The logo plots represent the conversation of epitopes for these three antibodies from 18 SARS-CoV-2, including WT, VOCs (Alpha, Beta, Gamma, Delta, Omicron), VOIs (Lambda, Mu) and other variants (Delta plus, Eta, Lota, Kappa, Theta, Iota, B.1.1.318, B.1.620, C.1.2 and C.363). **e** Superimposition of XGv264 and XGv286 upon SARS-CoV-2 Omicron S trimer and RBD in complex with XGv051, respectively. Color scheme is the same as in **b**. **f** Examination of lung and trachea tissues of mice challenged with Omicron collected at 5 dpi for virus titer. Virus RNA loads at 5 dpi were measured by RT-qPCR and are expressed as RNA copies per gram.

To delineate the structural basis for broad neutralization mediated by these NAbs, we determined the structures of a prefusion stabilized Omicron S trimer in complex with the Fab fragments of the three NAbs by cryo-electron microscopy (cryo-EM). We obtained cryo-EM reconstructions of these complexes at 3.0–3.8 Å and performed local refinement to further improve the densities around the binding interface between RBD and antibodies, enabling reliable analysis of the mode of interaction (Fig. 1b, Supplementary information, Figs. S3–S5 and Table S1). Unlike structural studies of the apo SARS-CoV-2 Omicron S trimer with a major configuration corresponding to one ‘up’ RBD and the other two RBDs in ‘down’ states,^3,8^ the XGv051-Omicron S complex structure revealed the S trimer adopting only one conformational state with all three RBDs in the ‘up’ state. In the structures of the Omicron S trimer bound with the other two antibodies, the S is observed assuming a configuration with two ‘up’ RBD and one ‘down’ RBD. These structural observations suggest that the binding of the three NAbs can result in a change in the conformational states of RBD, akin to ACE2 receptor, modulating/interfering with viral attachment (Fig. 1b).

Antibody XGv051 binds to an epitope at the epical tip of RBD, largely overlapping with the patch targeted by ACE2 (Fig. 1b; Supplementary information, Fig. S6). Strikingly, ten of eleven residues (except for G485) comprising the XGv051 epitope are implicated in extensive interactions with ACE2 (Supplementary information, Fig. S6). Tight binding for XGv051 is achieved by a network of hydrophobic interactions formed by Y449, Y453, L455, A475, A484, F486 and Y489 from Omicron RBD, I93 and Y94 from the light chain, G50, I52, F55, Y102, F104, H106, Y109 from the heavy chain and hydrophilic interactions, including 5 hydrogen bonds (Fig. 1c; Supplementary information, Table. S2). Structural comparisons revealed that XGv051 is very similar to S2K146 (Fig. 1b; Supplementary information, Fig. S6), an ultrapotent and broadly reactive NAb effective against all circulating SARS-CoV-2 variants,^9^ presumably cross neutralizing other sarbecoviruses through ACE2 molecular mimicry. Furthermore, XGv051 shows similar binding modes to those of A23-58.1 and XGv347 with slightly different orientations, and some other broadly cross-neutralizing antibodies (Supplementary information, Fig. S6).^3,10^ Of note, these NAbs belong to class II antibodies based on cluster analysis on epitope from 273 available RBD-NAb complex structures (Supplementary information, Fig. S7),^7^ revealing the presence of a subset of antibodies in class II being broadly potent against diverse SARS-CoV-2 variants.

Distinct from XGv051, the NAbs XGv264 and XGv286 bind patches surrounding the right shoulder of RBD with a similar binding mode and the binding region is broadly similar in locations to those observed for LY-CoV1404, XGv289 and XGv265, antibodies known to potently neutralize most VOCs (Fig. 1b, c; Supplementary information, Fig. S8).^3^ Specially, structures of XGv286 and XGv264 resemble those of XGv289 and XGv265, respectively (Supplementary information, Fig. S8). Compared to XGv265, XGv264 established two additional hydrogen bonds formed by N30 and E103 from heavy chain and T345 and K440 from RBD, respectively (Supplementary information, Fig. S9), conferring a considerably bigger surface area on RBD (850 Å^2^ vs 700 Å^2^ buried by XGv265 as described above), rationalizing improved binding and neutralizing activities against Omicron (Fig. 1a).^3^ Similarly, XGv289 and XGv286 shared almost same epitope residues, revealing conserved interactions at the interface for their high neutralizing potency (Supplementary information, Fig. S9). Both XGv264 and XGv286 paratopes constituted five complementarity determining regions (CDRs) with heavy chain and light chain contributing ~60% and ~40% of the binding surface area, respectively (Fig. 1c). Extensive hydrophobic and hydrophilic interactions facilitate the strong binding activities to Omicron (Fig. 1c). Epitope clustering analysis revealed that XGv264, XGv286 and other NAbs targeting the right shoulder of RBD constitute Class IV antibodies (Supplementary information, Fig. S7), indicating another broadly cross-reactive group, but with declined, to varying degrees, binding and neutralizing activities against Omicron due to the presence of new N440K and G446S mutations.^8^ These structural comparisons suggest that these NAbs might bind to SARS-CoV-2 variants in a homologous interaction manner (Fig. 1d), consistent with the analysis of RBD sequence conservation that showed a high degree of convergence of these epitopes, thereby defining RBD conserved sites of vulnerability in sarbecoviruses. In addition, structural superimpositions reveal that XGv051 and either XGv286 or XGv264 can simultaneously bind to S trimer, informing strategies to rationally design two-antibody cocktails (Fig. 1e).

To find out whether the observed in vitro neutralization potential confers in vivo protection, we tested one representative NAb, XGv051, in an established mouse model. Two groups of 9-month-old female K18-hACE2 transgenic mice were infected with 1 × 10^4^ PFU of SARS-CoV-2 Omicron BA.1, followed by intraperitoneal administration with a single dose of XGv051 at 20 mg per kilogram of body weight or PBS as a control at 2 hours after infection. As expected, robust virus replication with high levels of viral RNAs was detected in the lungs and trachea at 5 days post infection (dpi) in the control group of mice (Fig. 1f). Remarkably, a single dose of XGv051 administered resulted in > 99.9% reduction of the viral RNA loads in the lungs and a complete clearance of viruses in the trachea (Fig. 1f).

Our structural and functional analyses here revealed that broadly cross-reactive and ultrapotent antibodies generally meet the following criteria: (1) targeting key epitopes for efficient blockage of ACE2 recognition, (2) possessing extremely high binding affinities with *K*_*D*_ values of < 0.5 nM to WT RBD, (3) largely tolerating key epitope residue substitutions, (4) binding affinities to the variants are at least tenfold higher than those to ACE2. Results of our studies open up new avenues for developing therapeutics for combating existing and emerging variants to end the pandemic.

## Supplementary information

Supplementary Information

## Data Availability

The atomic coordinates of XGv051, XGv264 and XGv286 in complex with S trimer have been deposited in the protein data bank under accession codes 7WTF, 7WTI and 7WTK, respectively. Cryo-EM density maps for these complexes have been deposited at the Electron Microscopy Data Bank with accession codes EMD-32784 (XGv051), EMD-32787 (XGv264) and EMD-32789 (XGv286). The related atomic models and EM density maps of optimized reconstructions of Fab binding interface have been deposited under accession codes 7WTG (XGv051), 7WTH (XGv264), 7WTJ (XGv286), EMD-32785 (XGv051), EMD-32786 (XGv264) and EMD-32788 (XGv286), respectively.
