# Supplementary material for: Selection and structural bases of potent broadly neutralizing antibodies from 3-dose vaccinees that are highly effective against diverse SARS-CoV-2 variants, including Omicron sublineages
Source: Cell Res. 2022 Jun 7;32(7):691–4. doi: 10.1038/s41422-022-00677-z (PMC9171090; doi:10.1038/s41422-022-00677-z)
Supplement: Supplementary file 1 — Supplementary Information [file 41422_2022_677_MOESM1_ESM.pdf]

Supplementary information

Methods and Materials

Facility and ethics statements

The study protocol was approved by the Ethics Committee(seal) of Beijing Youan Hospital, Capital Medical University with an approval number of LL-2021-042-K. All participants provided written informed consent. All animal procedures were approved by the Institutional Animal Care and Use Committee of the Institute of Laboratory Animal Science, Peking Union Medical College (ILAS, PUMC) (No. DW21007). All experiments were performed in an animal biosafety level 3 (ABSL3) facility with high-efficiency particulate air (HEPA)–filtered isolators.

Viral stocks and Cell lines

SARS-CoV-2 WT strain CN01 was originally isolated from a patient in China during the early phase of COVID-19 endemic. The SARS-CoV-2 variants of concern (VOC) Beta (B.1.351 lineage) was isolated in a patient from South Africa; VOC gamma (P.1 lineage) was isolated from a person in Brazil; VOC delta (B.1.617.2 lineage) was isolated from a traveler infected back from India in early June and an Omicron (B.1.1.529 lineage) strain was isolated from a patient in Hong Kong and now preserved in SinoVac Biotech Ltd. All virus strains were first purified by standard plaque assay as previously described [1] and then inoculated into Vero cells (CCL-81) grown to 95% in 10% fetal bovine serum (FBS) supplemented Dulbecco's minimal essential medium (DMEM) for amplification. The cultures were maintained at 37 ℃ in an incubator supplied with 5% CO_2.

Single memory B cell isolation and sequencing

PBMCs were separated from the whole-blood samples obtained from four volunteers receiving three doses of CoronaVac inactivated vaccines by using Histopaque (Sigma) gradient centrifugation. Then the cells were isolated, aliquoted and stored in liquid nitrogen in the presence of FBS and DMSO. For single memory B cell sorting, stored PBMCs were thawed and incubated with CD19 MicroBeads (Miltenyi Biotec) to screen out CD19+ B lymphocytes, which were then incubated with human Fc block (BD Biosciences), anti-CD20-PECy7 (BD Biosciences), S-ECD-PE, and S-ECD-APC. The single memory B cells (CD20-1114 PECy7+ S-ECD-PE+ S ECD-APC+) were then sorted into 96-well plates using a FACSAria II (BD Biosciences), and followed by antibody sequencing and cloning as previously described.[2]

Antibody expression and Fab generation

The selected antibodies were subjected to vector construction and antibody expression as reported

previously.[3] Briefly, all the cloned human monoclonal antibodies were prepared by transient transfection into mammalian HEK293F cells, which were incubated for 5 days at 37°C under 5% CO_2 with shaking at 100 rpm for antibody expression. To generate the Fab fragments, the purified NAbs XGv051, XGv264 and XGv286 were processed using the Pierce FAB preparation kit (Thermo Scientific) as described previously.[4] Briefly, the samples were first applied to desalination columns to remove the salt, then the flow through was collected and incubated with papain that was attached with beads to cleave Fab fragments from the whole antibodies for 5 hours at 37°C. After that, the mixtures were transferred into Protein A affinity columns which specifically binds the Fc fragments. After centrifugation, the Fab fragments were collected and dialyzed into PBS (ThermoFisher, catalog #10010023).

Authentic virus neutralization assay

The monoclonal antibodies (mAbs) were diluted gradient from 50 μg/ml in two-fold steps and mixed with a virus suspension, which containing 100 $TCID_{50}$ at 36.5 ℃ for 2h. After that, the mixtures were added to wells seeded with confluence Vero cells and incubated at 36.5 ℃ for 5 days in a humidified 5% CO_2 cell incubator. Then the cytopathic effect (CPE) of each well was observed under microscopes by three different individuals. All the related dilutions and concentrations were recorded and used for the titration of samples tested by Reed-Muench method.

Pseudovirus neutralization assay

The pseudotyped viruses bearing the S protein were generated, aliquoted and restored as previous. 293T cell were first transfected with the plasmid embedded with the S gene of WT or VOCs (Alpha, Beta, Gamma, Delta and Omicron) SARS-CoV-2. The transfected 293T cells were infected with VSV G pseudotyped virus (G*ΔG-VSV) at a multiplicity of infection (MOI) of 4. After incubation for five hours, wash cell with PBS and add complete culture medium. After 24 hours, the SARS-CoV-2 pseudoviruses were produced and harvested. For the *In vitro* pseudotyped virus neutralization assay, the antibody samples were diluted in DMEM starting from 10 μg/ml with 10 additional threefold serial dilutions. Each of the sample were mixed with the SARS-CoV-2 pseudoviruses and incubated for 1h at 37 ℃. Then the mixture was added into Huh-7 cells and incubate for another 24h. The luciferase luminescence (RLU) of each well was measured with a luminescence microplate reader. The neutralization percentage was calculated as following: Inhibition (%) = [1- (sample RLU- Blank RLU) / (Positive Control RLU-Blank RLU)] (%). Antibody neutralization titers were presented as 50% maximal inhibitory concentration (IC_{50}).

Protein expression and purification

The sequence of VOC Omicron S protein (residues 1-1208) and receptor-binding domain (RBD) (residues 319-541) was mutated from the plasmids encoding the S and RBD of WT SARS-CoV-2 (GenBank: MN908947) in our lab by overlapping PCR. The proline substitutions at 817, 892, 899, 942, 986 and 987, 'GSAS' substitutions at the S1/S2 furin cleavage site (residues 682-685) and a C-terminal T4 foldon trimerization domain were also added into Omicron S construct to stabilize the trimeric conformation of S protein, in addition to the mutated residues reported (A67V, Δ69-70, T95I, G142D, Δ143-145, Δ211, L212I, ins214EPE, G339D, S371L, S373P, S375F, K417N, N440K, G446S, S477N, T478K, E484A, Q493R, G496S, Q498R, N501Y, Y505H, T547K, D614G, H655Y, N679K, P681H, N764K, D796Y, N856K, Q954H, N969K, L981F). To express the S protein, the plasmids of these proteins were transiently transfected into HEK293 F cells. The HEK293 F cells were grown in suspension at 37 °C in an incubator supplied with 8% CO_2, rotating at 130 rpm and harvested and concentrated three days post-transfection. The proteins were further purified by affinity chromatography using resin attached with streptavidin or Ni-NTA and size-exclusion chromatography (SEC) using a Superdex 200 column (GE Healthcare Life Sciences) with the buffer containing 20 mM Tris pH 8.0 and 200 mM NaCl.

Bio-layer interferometry

Bio-layer interferometry (BLI) experiments were run on an Octet Red 384 machine (Fortebio). To measure the binding affinities of mAbs, monoclonal antibodies were immobilized onto Protein A biosensors (Fortebio) and the threefold serial dilutions of WT RBD, Alpha RBD (ACROBiosystems, Cat No. SPD-C52Hn), Beta RBD (ACROBiosystems, Cat No. SPD-C52Hp), Gamma RBD (ACROBiosystems, Cat No. SPD-C52Hr), Delta RBD (ACROBiosystems, Cat No. SPD-C52Hh) and Omicron RBD (ACROBiosystems, Cat No. SPD-C522e) were used as analytes. Data were then analyzed using software Octet BLI Analysis 12.2 (Fortebio) with a 1:1 fitting model.

Cryo-EM sample preparation and data collection

The purified Omicron S trimer was mixed with XGv051, XGv264 and XGv286 Fab fragment with a molar ratio of 1: 1.2 for 10s ice incubation, respectively. Then, 3μl of S trimer - Fab complexes were dropped onto the pre-glow-discharged gold grid (C-flat, 300-mesh, 1.2/1.3, Protochips In.), blotted for 6 seconds with no force in 100% relative humidity and immediately plunged into the liquid ethane using Vitrobot (FEI). Cryo-EM data were collected by at 300 kV with an FEI Titan Krios microscope (FEI). Movies of Omicron S-XGv051 complex (32 frames, each 0.2 s, total dose

of 40 e^{-} $Å^{-2}$) were recorded using a K2 Summit direct detector with a defocus range between 1.2-1.8 µm, while movies of Omicron S-XGv264 or XGv286 complex (32 frames, each 0.2 s, total dose of 60 e^{-} $Å^{-2}$) were recorded using a K3 Summit direct detector with a defocus range between 1.5-2.5 µm. Automated single particle data acquisition was carried out by SerialEM, with a calibrated magnification of 22,500 yielding a final pixel size of 1.04 or 1.07 Å.

Cryo-EM data processing

A total of 3,803, 4,890 and 4,992 movies of S-XGv051, S-XGv264 and S-XGv286 complexes were recorded and subjected to beam-induced motion correction using MotionCorr in RELION 3.0 package. Then the defocus value of each motion corrected micrograph was estimated by Gctf. 347,106, 2,815,543 and 2,840,573 particles were autopicked and extracted for 2D Classification by cryoSPARC and 3D Classification by RELION. After that, 126,909, 494,849 and 339,195 particles were selected and processed by non-uniform auto-refinement and postprocessing in cryoSPARC to generate the final cryo-EM density. Local refinement was performed to further improve the resolution of the binding interface of these three complexes. The resolution of each structure was determined based on the gold-standard Fourier shell correlation (threshold = 0.143) and evaluated by ResMap.

Model fitting and refinement

The atomic models of the complexes were generated by first fitting the chains of the native apo SARS-CoV-2 S trimer (PDB number of 6VYB) and Fabs (PDB number of 7LSS and 5UR0 for XGv051, 7R8O and 5GZO for XGv264, 6UDA and 6A67 for XGv286) into the cryo-EM densities of the final complexes by Chimera, followed by manually adjustment and correction according to the protein sequences and densities in Coot, as well as real space refinement using Phenix. The final models were evaluated by Molprobity.

Protection against SARS-CoV-2 Omicron variant strain challenge in mice

The in vivo protection efficacy of XGv051 antibody was assessed by using a mouse model challenged with SARS-CoV-2 Omicron variant strain. The SARS-CoV-2 virus was designated as SARS-CoV-2/human/CHN/Omicron-1/2021 (Genbank: OM095411.1) were provided by ILAS, PUMC, China. Two groups of 9-month-old K18-hACE2 mice (provided by GemPharmatech Co., Ltd.) were infected with 1×10^{4} PFU of Omicron strain, then infected mice were treated intraperitoneally with XGv051 antibody or PBS as a control at 2 hours after infection. The lung and trachea tissues of these mice were collected at 5 dpi for viral RNA loads assay. Tissues homogenates

were clarified by centrifugation and viral RNA was extracted using the QIAamp Viral RNA Mini Kit (Qiagen). Viral sgRNA quantification was measured by quantitative reverse transcription PCR (RT-qPCR) targeting the S gene of SARS-CoV-2. RT-qPCR was performed using One-Step PrimeScript RT-PCR Kit (Takara).

Quantification RT-PCR

Total RNA was extracted and reverse transcription was performed as described previously (1). Briefly, qRT–PCR was carried out using the following cycling protocol and primers: 50°C for 2 min, then 95°C for 2 min, followed by 40 cycles of 95°C for 15 s and 60°C for 30 s, and final incubations at 95°C for 15 s, 60°C for 1 min, and 95°C for 45 s. The following primers were used to detect SARS-CoV-2: SARS-CoV-2: Forward primer, 5′-TCGTTTCGGAAGAGACAGGT-3′; Reverse primer, 5′-GCGCAGTAAGGATGGCTAGT-3′.

References

1. Gao, Q. *et al. Science* **369**, 77-81 (2020).
2. Zhou, Y. *et al. Cell reports* **34**, 108699 (2021).
3. Wang, K. *et al. medRxiv* (2021).
4. Wang, N. *et al. Cell research* **31**, 101-103 (2021).

Supplementary Figures

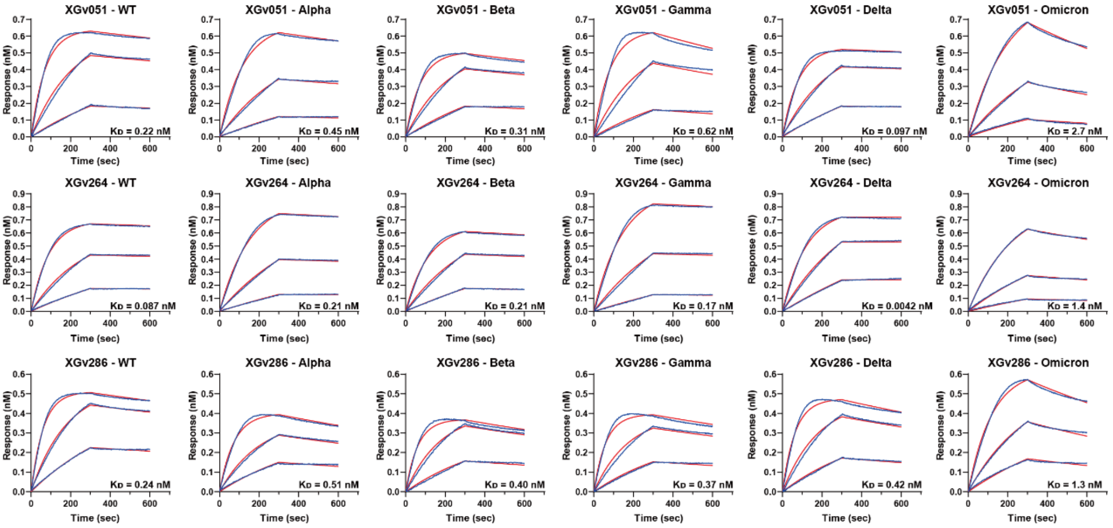

Supplementary information, Fig. S1 The binding Affinities of XGv051, XGv264 and XGv286 to SARS-CoV-2 Omicron RBD. Binding curves of all XGv051, XGv264 and XGv286 against SARS-CoV-2 Omicron RBD were measured by biolayer interferometry (BLI).

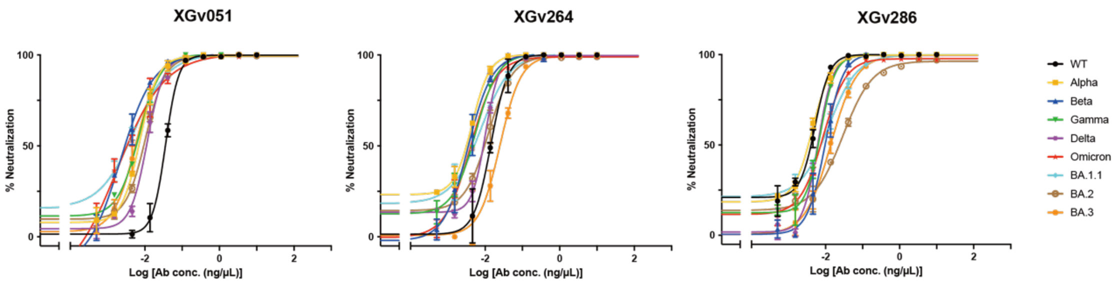

Supplementary information, Fig. S2 Neutralization curves of XGV051, XGv264 and XGv286 on pseudoviruses. Neutralization curves for XGv051, XGv264 and XGv286 pseudoviruses with the SARS-CoV-2 wild-type and VOCs.

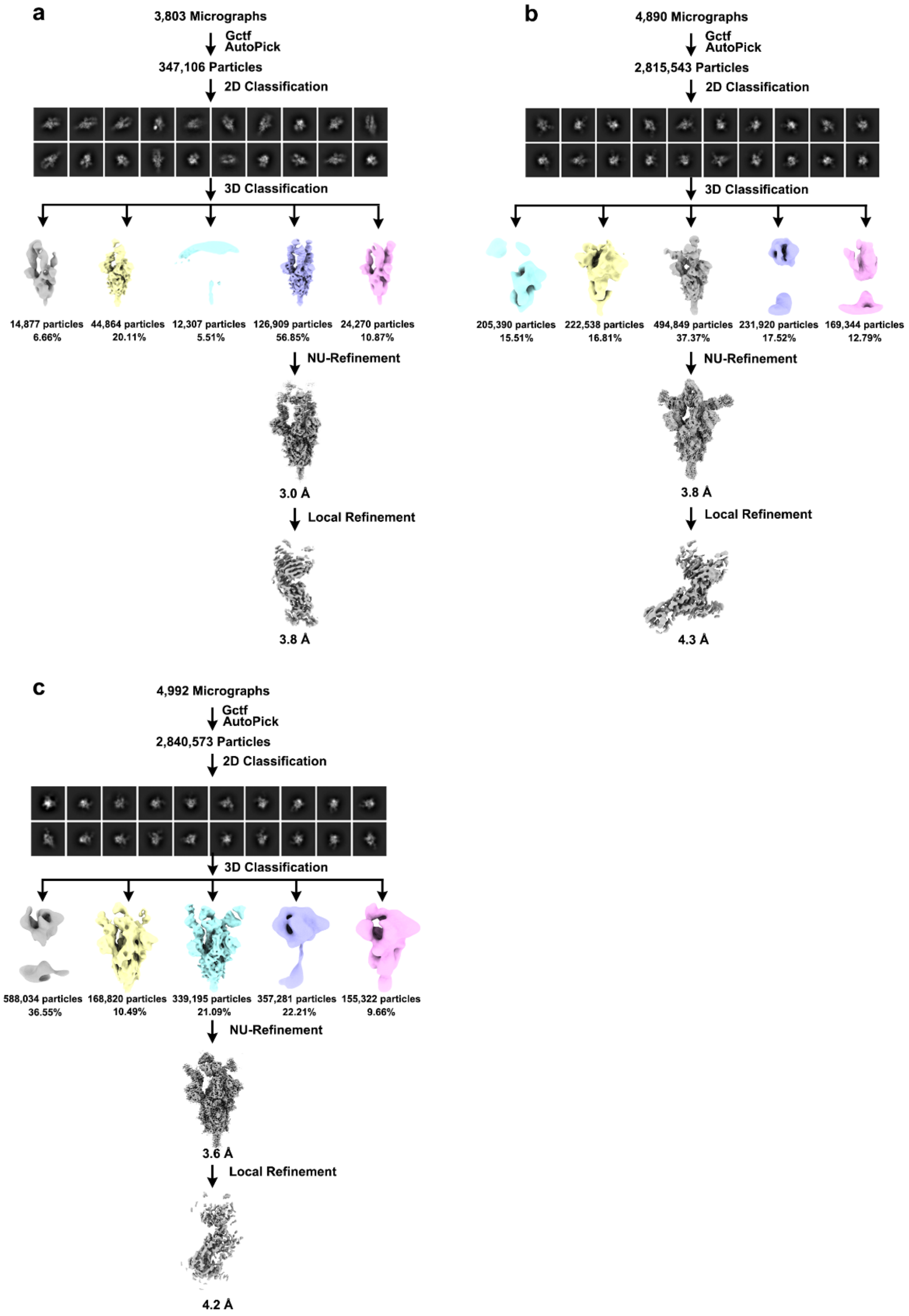

Supplementary information, Fig. S3 Flowcharts for data processing. Flowcharts for SARS-CoV-2 Omicron S trimer in complex with (a) XGv051, (b) XGv264 and (c) XGv286.

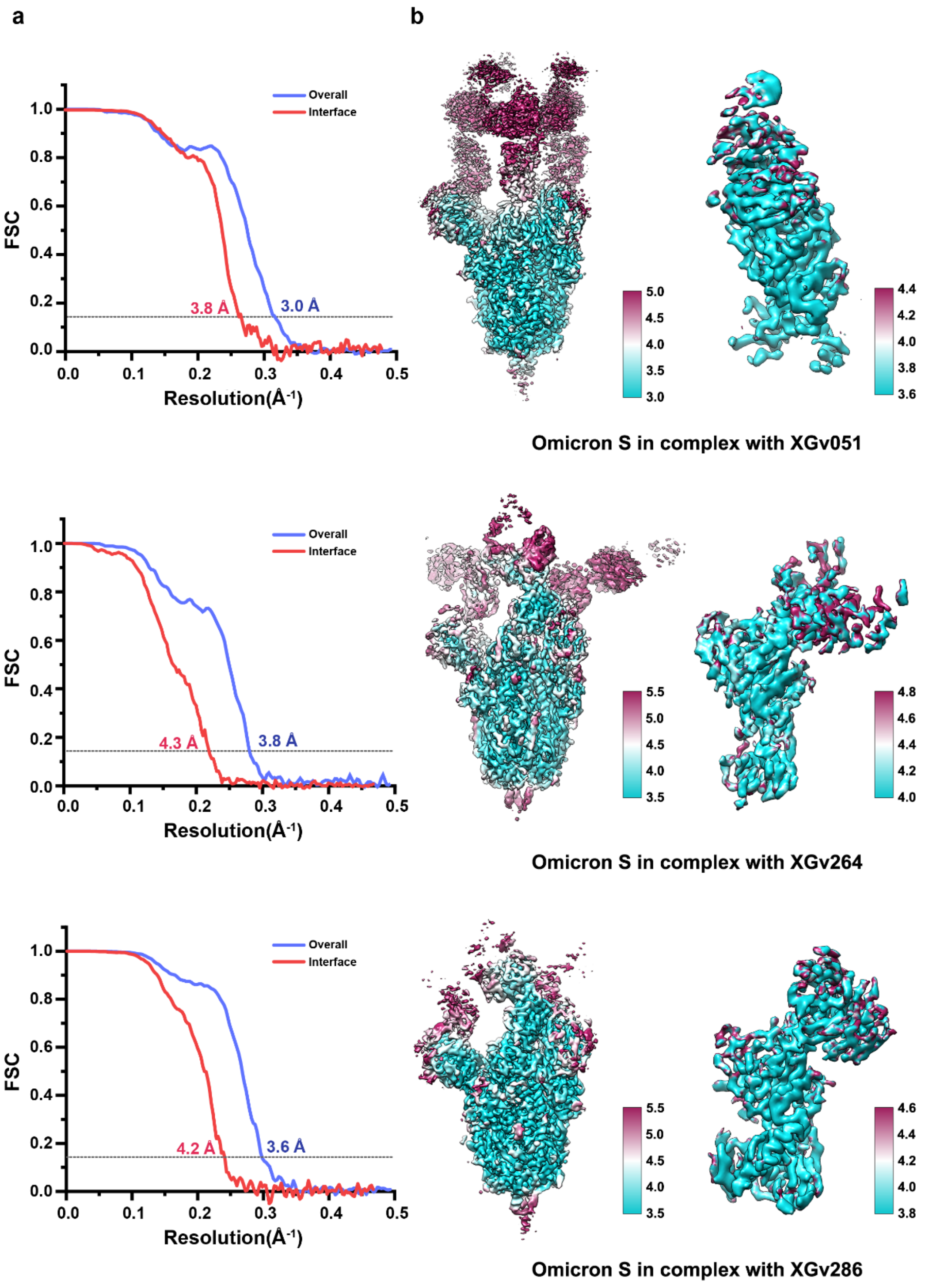

Supplementary information, Fig. S4 Resolution estimation of the EM maps. (a) The gold-standard FSC curves of overall maps of Omicron S trimer in complex with Fab XGv051, XGv264, XGv286 and local maps of interfaces. (b) Local resolution assessments of cryo-EM maps using ResMap are shown.

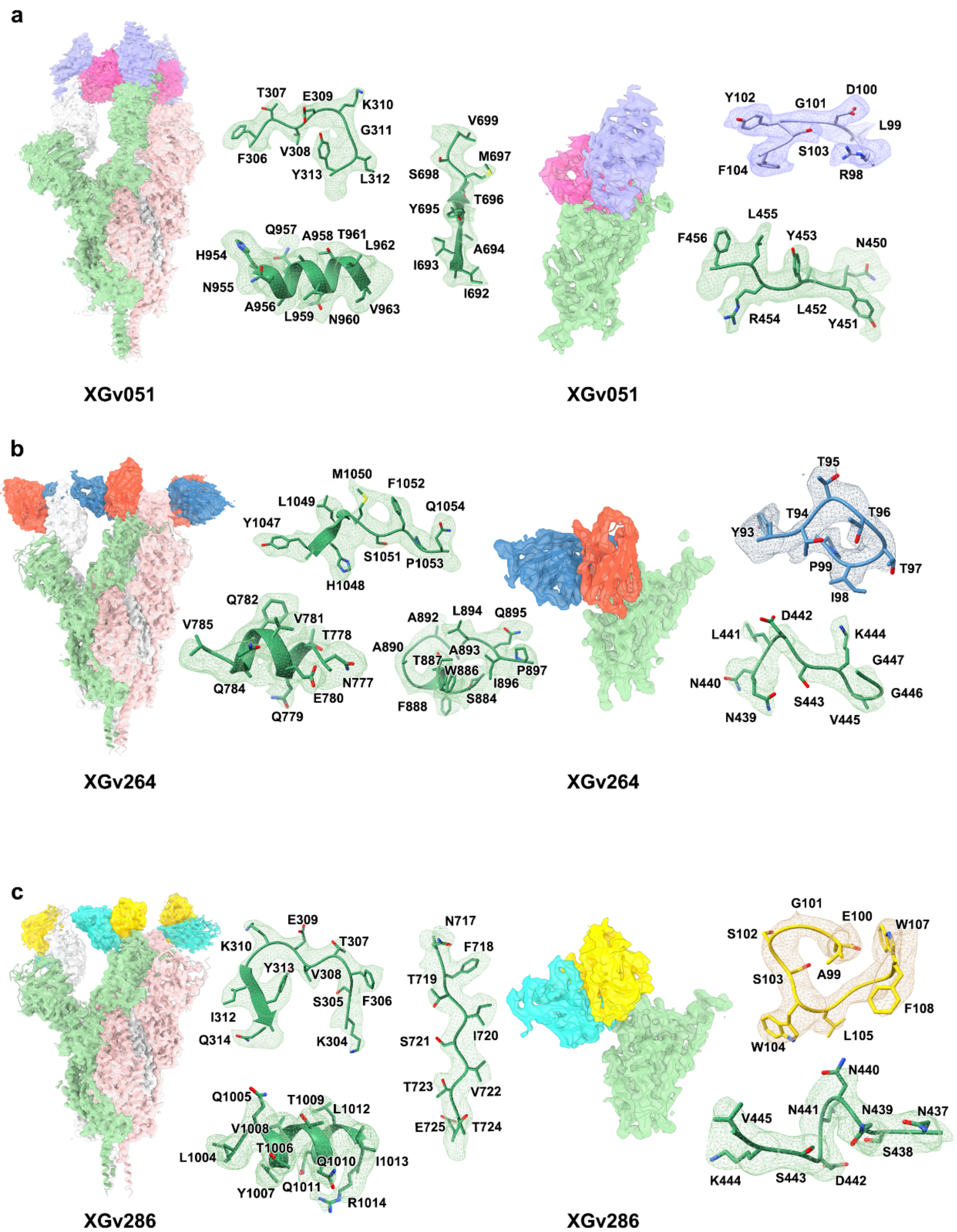

Supplementary information, Fig. S5 Density maps and atomics models. Cryo-EM density maps of Omicron S trimer in complex with XGv051, XGv264, XGv286 and their interfaces are shown. Color scheme is the same as in **Fig. 1**. Residues are shown as sticks with oxygen colored in red, nitrogen colored in blue and sulfurs colored in yellow.

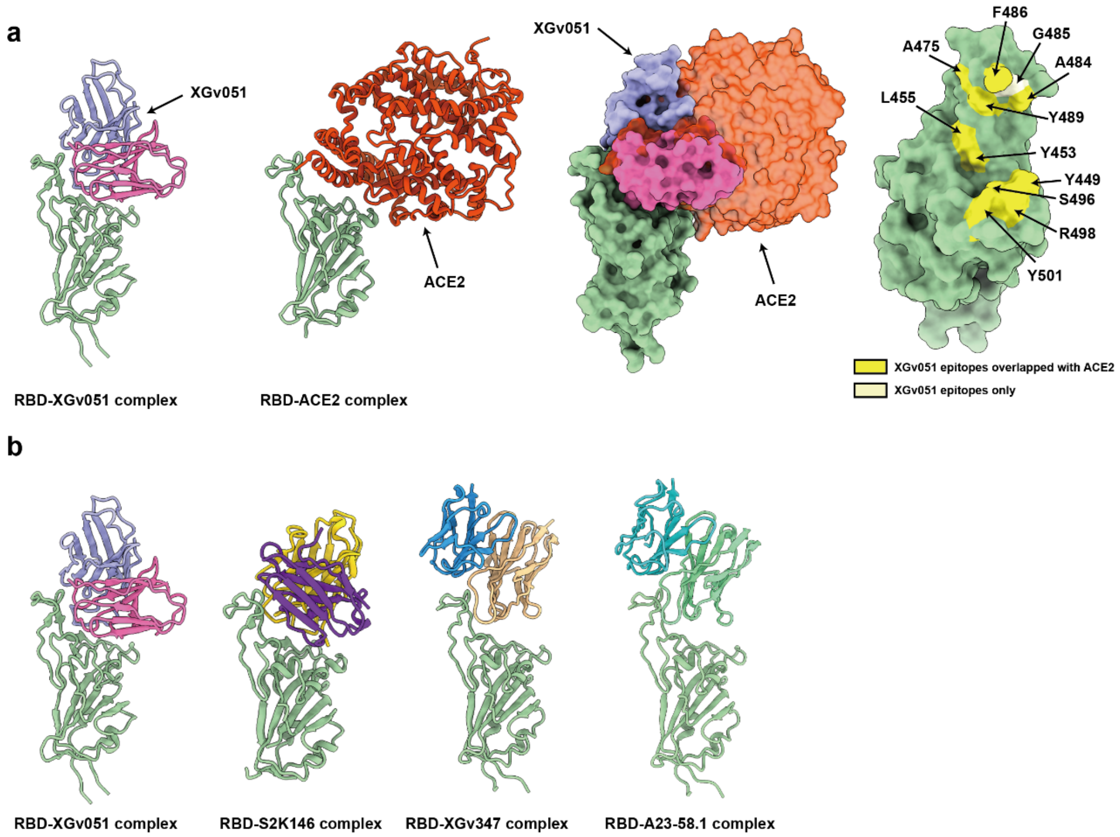

Supplementary information, Fig. S6 Binding mode of XGv051. (a) Surface representation of the structure of SARS-CoV-2 Omicron S trimer in complex with XGv051. The left panels show the clash between ACE2 and XGv051. RBD is colored in light green, ACE2 is colored in orange and the color scheme of XGv051 is the same as **Fig. 1**. The rightmost panel show surface representations of the SARS-CoV-2 Omicron RBD. Residues colored in yellow (pale and bright yellow) are the residues recognized by XGv051. Among these, residues overlapping with binding sites for ACE2 are colored in bright yellow. (b) Binding modes of XGv051, S2K146, XGv347 and A23-58.1. RBD is colored in light green. S2K146, XGv347, A23-58.1 is colored in purple and yellow, blue and light yellow, cyan and green. The color scheme of XGv051 is the same as **Fig. 1**.

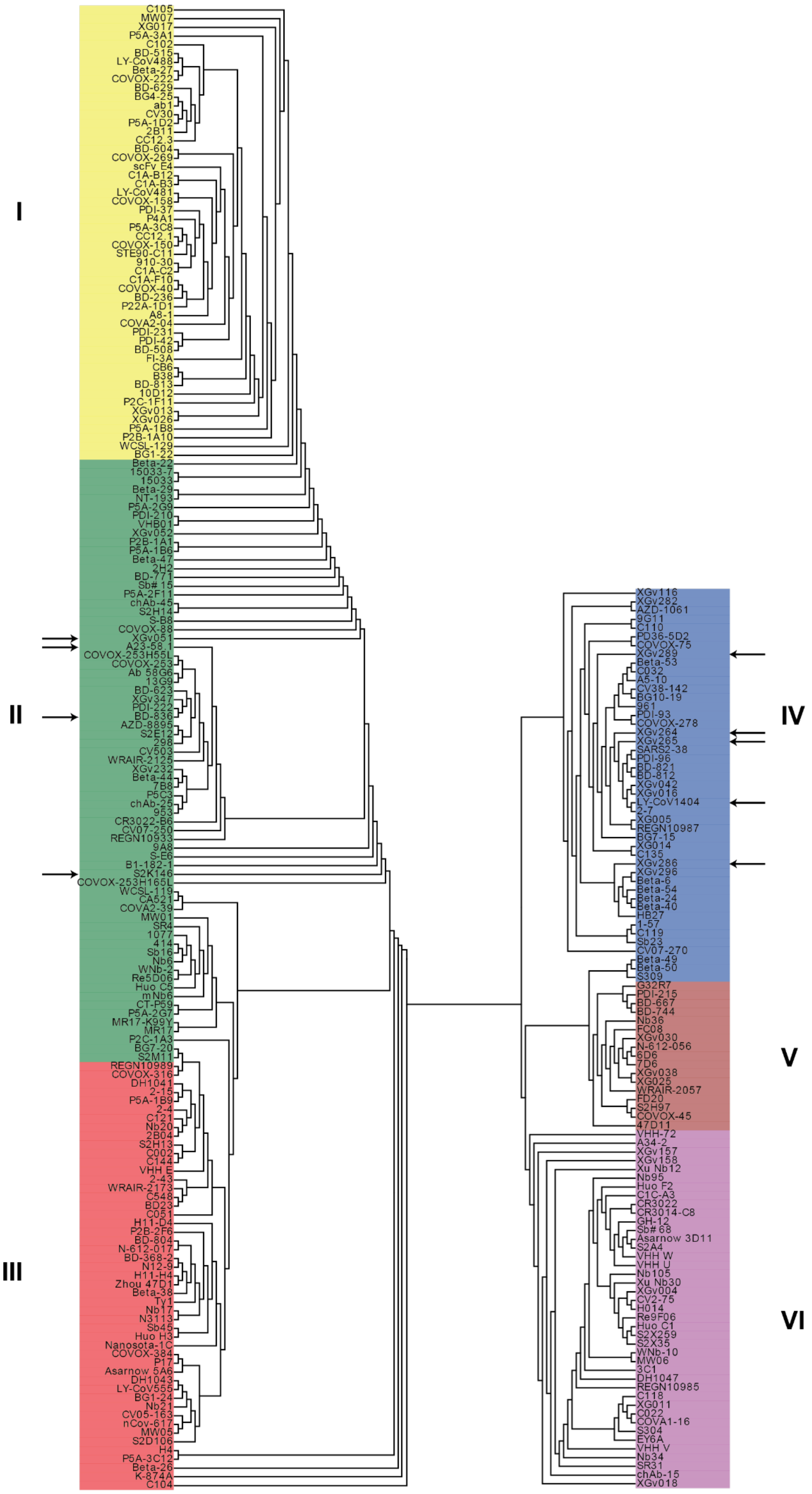

I
C105
MW07
XG017
P5A-3A1
C102
BD-515
LY-CoV488
Beta-27
COVOX-222
BD-629
BG4-25
ab1
CV30
P5A-1D2
2B11
CC12.3
BD-604
COVOX-269
scFv E4
C1A-B12
C1A-B3
LY-CoV481
COVOX-158
PDI-37
P4A1
P5A-3C8
CC12.1
COVOX-150
STE90-C11
910-30
C1A-C2
C1A-F10
COVOX-40
BD-236
P22A-1D1
A8-1
COVA2-04
PDI-231
PDI-42
BD-508
FI-3A
CB6
B38
BD-813
10D12
P2C-1F11
XGv013
XGv026
P5A-1B8
P2B-1A10
WCSL-129
BG1-22
II
Beta-22
15033-7
15033
Beta-29
NT-193
P5A-2G9
PDI-210
VHB01
XGv052
P2B-1A1
P5A-1B6
Beta-47
2H2
BD-771
Sb#15
P5A-2F11
chAb-45
S2H14
S-B8
COVOX-88
XGv051
A23-58.1
COVOX-253H55L
COVOX-253
Ab 58G6
13G9
BD-623
XGv347
PDI-222
BD-836
AZD-8895
S2E12
298
CV503
WRAIR-2125
XGv232
Beta-44
7B8
P5C3
chAb-25
953
CR3022-B6
CV07-250
REGN10933
9A8
S-E6
B1-182-1
S2K146
COVOX-253H165L
WCSL-119
CA521
COVA2-39
MW01
SR4
1077
414
Sb16
Nb6
WNb-2
Re5D06
Huo C5
mNb6
CT-P59
P5A-2G7
MR17-K99Y
MR17
P2C-1A3
BG7-20
S2M11
III
REGN10989
COVOX-316
DH1041
2-15
P5A-1B9
2-4
C121
Nb20
2B04
S2H13
C002
C144
VHH E
2-43
WRAIR-2173
C548
BD23
C051
H11-D4
P2B-2F6
BD-804
N-612-017
BD-368-2
N12-9
H11-H4
Zhou 47D1
Beta-38
Ty1
Nb17
N3113
Sb45
Huo H3
Nanosota-1C
COVOX-384
P17
Asarnow 5A6
DH1043
LY-CoV555
BG1-24
Nb21
CV05-163
nCov-617
MW05
S2D106
H4
P5A-3C12
Beta-26
K-874A
C104
IV
XGv116
XGv282
AZD-1061
9G11
C110
PD36-5D2
COVOX-75
XGv289
Beta-53
C032
A5-10
CV38-142
BG10-19
961
PDI-93
COVOX-278
XGv264
XGv265
SARS2-38
PDI-96
BD-821
BD-812
XGv042
XGv016
LY-CoV1404
2-7
XG005
REGN10987
BG7-15
XG014
C135
XGv286
XGv296
Beta-6
Beta-54
Beta-24
Beta-40
HB27
1-57
C119
Sb23
CV07-270
Beta-49
Beta-50
S309
V
G32R7
PDI-215
BD-667
BD-744
Nb36
FC08
XGv030
N-612-056
6D6
7D6
XGv038
XG025
WRAIR-2057
FD20
S2H97
COVOX-45
47D11
VI
VHH-72
A34-2
XGv157
XGv158
Xu Nb12
Nb95
Huo F2
C1C-A3
CR3022
CR3014-C8
GH-12
Sb#68
Asarnow 3D11
S2A4
VHH W
VHH U
Nb105
Xu Nb30
XGv004
CV2-75
H014
Re9F06
Huo C1
S2X259
S2X35
WNb-10
MW06
3C1
DH1047
REGN10985
C118
XG011
C022
COVA1-16
S304
EY6A
VHH V
Nb34
SR31
chAb-15
XGv018

Supplementary information, Fig. S7 Cluster analysis on epitope from 273 available RBD-NAb complex structures.

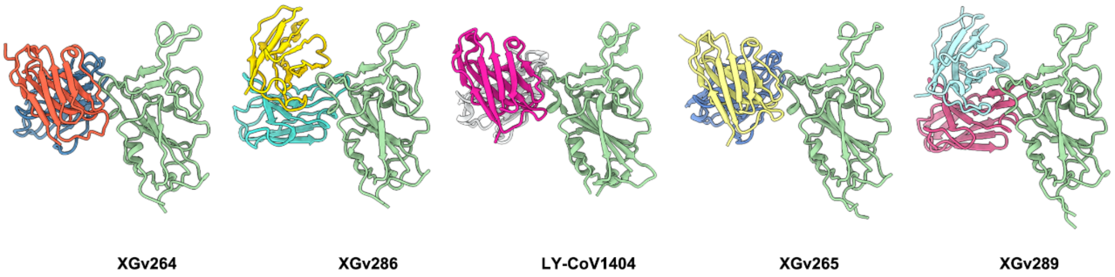

Supplementary information, Fig. S8 Structural comparison of XGv264, XGv286, LY-CoV1404, XGv265 and XGv289. RBD is colored in light green. LY-CoV1404, XGv265 and XGv289 is colored in magenta and white, light blue and light yellow, cyan and pink. The color scheme of XGv264 and XGv286 is the same as **Fig. 1**.

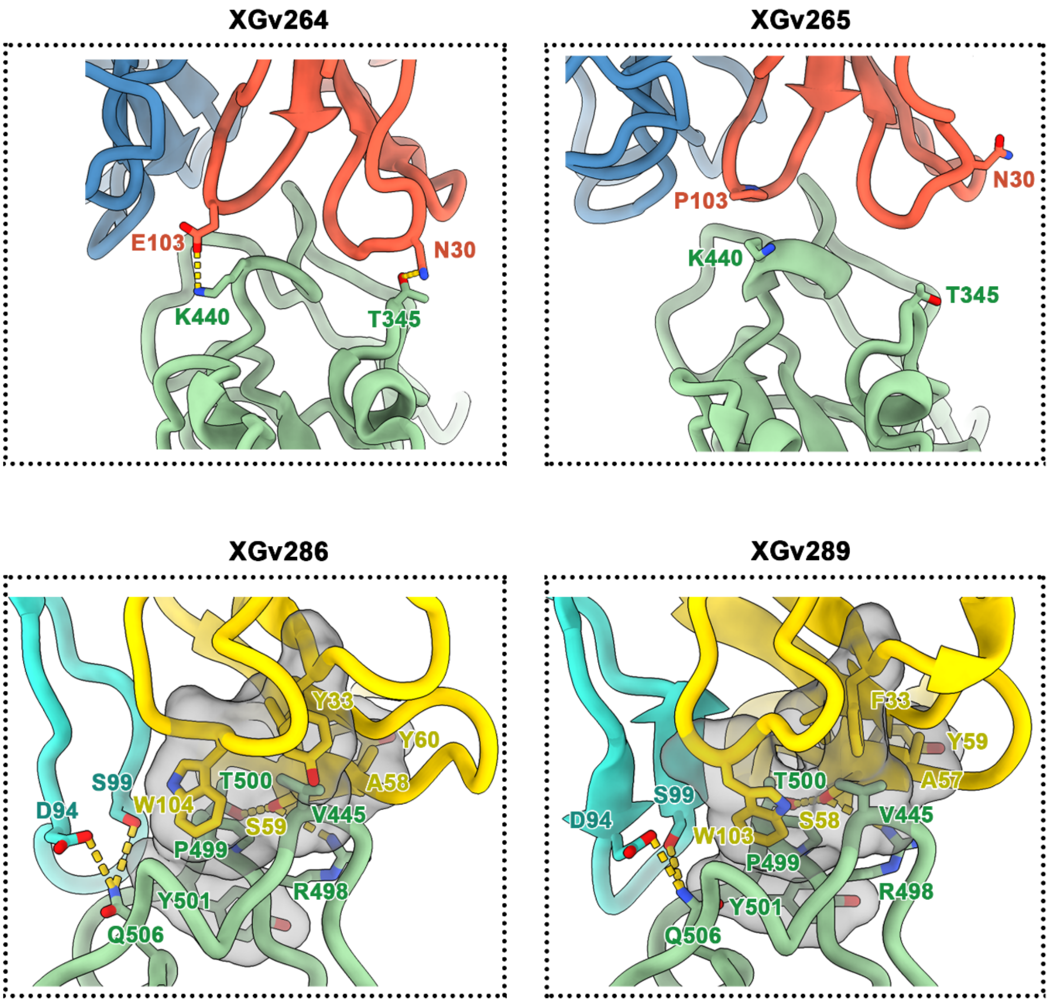

Supplementary information, Fig. S9 Details of Structural comparison of XGv264 and XGv265, XGv286 and XGv289. Comparison of XGv264 and XGv265 (Upper), XGv286 and XGv289 (lower) are shown as cartoon. Structural differences between XGv264 and XGv285 are shown. Conserved interactions at the binding interface of XGv286-RBD and XGv289-RBD are represented.

Supplementary information, Table S1 | Statistics for cryo-EM data collection, refinement, and validation

	Omicron S trimer in complex with XGv051	Omicron S trimer in complex with XGv264	Omicron S trimer in complex with XGv286	XGv051-RBD-interface	XGv264-RBD-interface	XGv286-RBD-interface
**Data collection and processing**						
Magnification	22,500	22,500	22,500	22,500	22,500	22,500
Voltage (kV)	300	300	300	300	300	300
Electron exposure (e–/Å^2)	60	60	60	60	60	60
Defocus range (μm)	-1.2--1.8	-1.5--2.5	-1.5--2.5	-1.2--1.8	-1.5--2.5	-1.5--2.5
Pixel size (Å)	1.04	1.07	1.07	1.04	1.07	1.04
Symmetry imposed	C1	C1	C1	C1	C1	C1
Initial particle images (no.)	347,106	2,815,543	2,840,573	347,106	2,815,543	2,840,573
Final particles images (no.)	126,909	494,849	339,195	380,727	494,849	339,195
Map resolution (Å)	3.0	3.8	3.6	3.8	4.3	4.2
FSC threshold	0.143	0.143	0.143	0.143	0.143	0.143
Map resolution range (Å)	3.0-60	3.8-60	3.6-60	3.8-60	4.3-60	4.2-60
**Refinement**						
Initial model used (PDB code)	7WEA	7WEA	7WEA	7WEA	7WEA	7WEA
Model resolution (Å)	3.3	3.3	3.3	3.3	3.3	3.3
FSC threshold	0.143	0.143	0.143	0.143	0.143	0.143
Model resolution range (Å)	3.3-60	3.3-60	3.3-60	3.3-60	3.3-60	3.3-60
Map sharpening *B* factor (Å^2)	103.2	157.4	167.9	237.3	285.7	298.9
Model composition						
Non-hydrogen atoms	3,2040	31,988	31,900	3,358	3,255	3,259
Protein residues	3,966	3,981	3,975	431	421	424
Ligands	75	70	71	0	0	0
B factors (Å^2)						
Protein	103.02	129.89	115.94	54.12	51.17	67.46
Ligand	109.35	182.63	148.89	-	-	-
R.m.s. deviations						
Bond lengths (Å)	0.004	0.004	0.004	0.006	0.003	0.003
Bond angles (°)	0.831	0.903	0.797	1.196	0.715	0.625
Validation						
MolProbity score	1.73	1.79	1.79	1.74	1.99	2.03
Clashscore	5.48	5.77	5.66	4.46	9.24	10.41

Poor rotamers (%)	0.00	0.03	0.03	0.00	0.00	0.00
Ramachandran plot						
Favored (%)	93.41	92.36	92.18	91.17	91.57	91.87
Allowed (%)	6.54	7.52	7.66	8.83	8.43	8.13
Disallowed (%)	0.05	0.13	0.15	0.00	0.00	0.00

Supplementary information, Table S2 | List of interacting residues between Fabs and Omicron SARS-CoV-2 S trimer (d < 4 Å)

Complex	Omicron RBD	Heavy chain	Light chain
**Omicron S-trimer in complex with XGv051**	Y449	H106	
	Y453	Q111	
	L455	Y109	
	A475		I93
	A484	F55 F104	
	G485	F104	T99
	F486	G50 I52	Y94
	Y489	Y102	Y94
	S496	R108	
	R498	R108	
	Y501	R108	
**Omicron S-trimer in complex with XGv264**	T345	N30	
	R346	D56	
	N439		Y34
	N440	E103	Y34
	L441	G32 G33	
	K444	Y54	
	V445	L52 Y54	P99
	N450	D58	
	P499		Y34 Y93
	T500		Y93 T94 T95
**Omicron S-trimer in complex with XGv286**	V445	Y33 50I 58A 104W	
	R498	A58 S59	
	P499	W104	
	T500	S59 Y60	S99
	Y501		S99
	V503		L96
	Q506		D94

Supplementary information, Table S3 | Amino acid variations of pseudoviruses

	Amino acid variations
**Alpha**	69-70del, 144del, N501Y, A570D, D614G, P681H, T716I, S982A, D1118H
**Beta**	241-243del, D80A, D215G, K417N, E484K, N501Y, D614G, A701V
**Gamma**	L18F, T20N, P26S, D138Y, R190S, K417T, E484K, N501Y, D614G, H655Y, T1027I, V1176F
**Delta**	157-158del, T19R, E156G, L452R, T478K, D614G, P681R, D950N
**Omicron (BA.1)**	69-70del, 143-145del, 212del, ins214EPE, A67V, T95I, G142D, N211I, G339D, S371L, S373P, S375F, K417N, N440K, G446S, S477N, T478K, E484A, Q493R, Q496S, Q498R, N501Y, Y505H, T547K, D614G, H655Y, N679K, P681H, N764K, D796Y, N856K, Q954H, N969K, L981F
**BA.1.1**	69-70del, 143-145del, 212del, ins214EPE, A67V, T95I, G142D, N211I, G339D, R346K, S371L, S373P, S375F, K417N, N440K, G446S, S477N, T478K, E484A, Q493R, Q496S, Q498R, N501Y, Y505H, T547K, D614G, H655Y, N679K, P681H, N764K, D796Y, N856K, Q954H, N969K, L981F
**BA.2**	25-27del, T19I, L24S, G142D, V213G, G339D, S371F, S373P, S375F, T376A, D405N, R408S, K417N, N440K, S477N, T478K, E484A, Q493R, Q498R, N501Y, Y505H, D614G, H655Y, N679K, P681H, N764K, D796Y, Q954H, N969K
**BA.3**	69-70del, 143-145del, 212del, A67V, T95I, G142D, N211I, V213G, G339D, S371F, S373P, S375F, D405N, K417N, N440K, G446S, S477N, T478K, E484A, Q493R, Q498R, N501Y, Y505H, D614G, H655Y, N679K, P681H, N764K, D796Y, Q954H, N969K
